# Dietetic Progress

**Published:** 1891-06

**Authors:** 


### Dietetic Progress.

Astronomy means the laws of the stars; gastronomy means the laws of the gullet. A grand dinner is a complicated affair, and has only been evolved by ages of civilization and culture. Primitive races eat whatever they can get and whenever they can get it. Nomadic tribes, with flocks and herds, establish regular needs, and discover the oldest gastronomic combination, bread and meat. The peasantry of civilized nations combine dishes together to make the "square meal," say meat, potatoes and a relish with bread and butter. Cultured people gradually come to add two courses to this "square meal," one at the end called dessert to help digestion; the other at the beginning, the preliminary course or gusto, to whet the appetite. In a state of still higher refinement each of these two courses falls into two parts. The "after-meal" comprises pastry and dessert; the "mid-meal" meat and game; the "preliminary meal" soup and fish. But the height is reached in the grand symposium or banquet of nine courses: 1, hors d' ævre; 2, potage; 3, releve; 4 ———; 5, entree; 6, roti; 7, entremett; 8, sucree; 9, dessert. Nine courses exclusive of punches and coffee! Nine meals in one! Apres nons le deluge—indigestion, gout and biliousness; exit the cook, enter the doctor.—*Exchange.*

